# Validation of Recalibrated IOPVet in Canine Eyes Compared to Manometry and Recalibrated TONOVET Plus

**DOI:** 10.1111/vop.70100

**Published:** 2025-10-13

**Authors:** Bertrand Michaud, Inès Desquiens

**Affiliations:** ^1^ Clinique Vétérinaire Anima‐Vet Saint‐Genis‐Pouilly France

**Keywords:** canine, glaucoma, intraocular pressure, IOPVet, tonometry

## Abstract

**Objective:**

To evaluate the clinical and manometric accuracy of a recalibrated version of the IOPVet indentation tonometer in dogs, and to compare its performance to a recalibrated TonoVet Plus (rTVP) and in situ direct manometry.

**Animals Studied:**

A total of 97 eyes from 49 client‐owned dogs were enrolled and divided into three groups: a glaucomatous group of 34 dogs (67 eyes: 41 glaucomatous, 26 fellow normotensive eyes), a control group of 10 healthy dogs (20 eyes), and a manometric group of 10 eyes from 5 euthanized dogs.

**Procedures:**

IOP was measured using both the rTVP and the recalibrated IOPVet, each device being used by a separate examiner. IOPVet readings were recorded as semi‐quantitative intervals: < 10, 10–29, 30–49, and ≥ 50 mmHg. Agreement with rTVP and manometric values was assessed through sensitivity, specificity, and Chi‐square analysis. Manometry was performed post‐mortem using dual‐needle in situ cannulation over a range of pressures (10–70 mmHg).

**Results:**

All glaucomatous eyes (rTVP ≥ 30 mmHg, *n* = 41) were correctly classified by the IOPVet (sensitivity 100%). Among normotensive eyes (rTVP ≤ 29 mmHg, *n* = 46), 78.3% were correctly categorized, with 21.7% overestimated (specificity 84.8%). The association between rTVP and IOPVet categories was significant (χ^2^ = 50.2, df = 4, *p* < 0.0001). In manometric validation, accuracy was 95% (10–29 mmHg), 100% (30–49 mmHg), and 94% (≥ 50 mmHg), with no misclassification below 30 mmHg.

**Conclusions:**

The recalibrated IOPVet demonstrates markedly improved accuracy, excellent sensitivity, and acceptable specificity in detecting elevated IOP in dogs. Its affordability and ease of use support its integration into clinical practice and owner‐assisted glaucoma monitoring.

## 1 Introduction

Intraocular pressure (IOP) is a fundamental parameter in ocular physiology and pathology. Its elevation is strongly associated with the development and progression of glaucoma, a leading cause of irreversible blindness in dogs. The accurate and repeatable measurement of IOP is therefore essential for both the diagnosis and long‐term monitoring of patients affected by or at risk of glaucomatous disease [1, 2].

In veterinary ophthalmology, rebound tonometry is the gold standard non‐invasive method used to estimate IOP. A wide range of rebound tonometers have been validated for use in companion animals, including the TonoVet (TV), TonoVet Plus (TVP), recalibrated TonoVet Plus (rTVP), TonoVet Pro (TVPR), and TonoVera Vet (TVV). These devices are generally preferred over applanation or indentation systems due to their ease of use, reproducibility, and patient tolerance [3, 4]. They do not require any topical anesthesia, and their digital readouts provide efficient interpretation and data recording [3, 4]. The TVP and TVPR, in particular, have demonstrated strong agreement with direct manometry, including in the high IOP range relevant to glaucomatous pathology [3]. Nevertheless, the relatively high cost of these devices and the need for regular recalibration may limit their use in primary care veterinary clinics or for at‐home monitoring.

To address these limitations, the IOPVet (Ingeneus Pty. Ltd., Australia) has been introduced as a low‐cost, single‐use indentation tonometer intended to provide a simplified, color‐coded estimation of intraocular pressure. Originally developed for use in human medicine, the device appears to be identical to the Ingeneus eyePressure tonometer and was subsequently marketed for veterinary application without any prior validation in the animal species for which it is now recommended [1]. While its semi‐quantitative approach could theoretically facilitate screening or remote monitoring of IOP in pets, early investigations highlighted serious limitations in its capacity to detect elevated IOP with a tendency to underevaluate high pressures [1, 2].

In clinical settings, sensitivity dropped to 67.9% for IOPs between 20 and 30 mmHg and plummeted to 33.3% for pressures > 30 mmHg in Michaud's study and even less with just 13% for glaucomatous eyes in Kapeller's report, with most elevated pressures being underestimated. These findings strongly suggest that the device, in its current form, is unsuitable for clinical decision‐making in canine glaucoma management, leading to a huge number of false‐negative cases.

**FIGURE 1:**
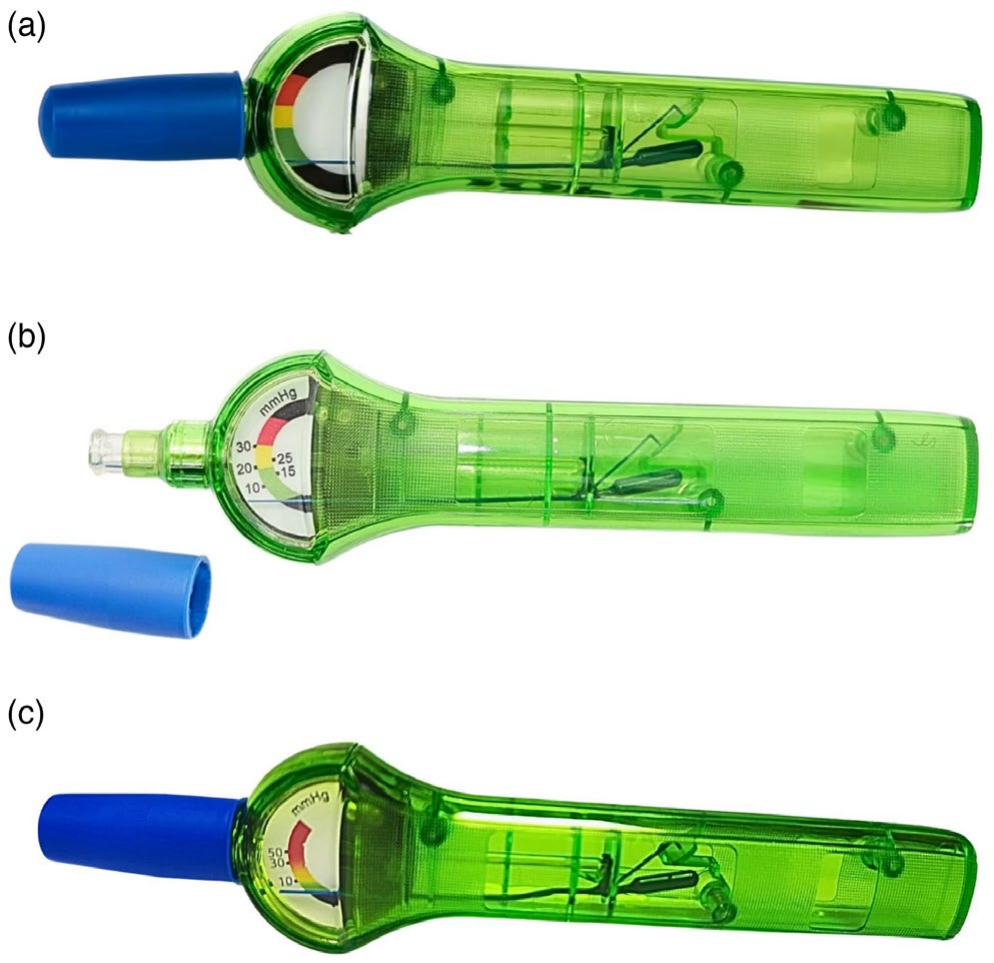
The three versions of the IOPVet: The first version (a), without numerical IOP indications, was evaluated in the study by Kapeller. The second version (b), featuring multiple numerical IOP indications, was assessed in the study by Michaud. Both of these versions display an abrupt color change from one color to another. Finally, the third version (c), which was evaluated in our study: The upper black range disappears, the color scale is modified to become progressive, and the numerical indications are limited to three pressure values: 10, 30, and 50 mmHg.

Following these studies, we submitted these observations to the Ingeneus company and proposed a revision of the scale used by the device. The original version of the IOPVet device, as used in the studies by Kapeller and Michaud, featured a color‐coded scale with sharply defined zones: green (< 20 mmHg), yellow (20–30 mmHg), and red (> 30 mmHg), along with two black zones (one below and one above the color range) that had not been associated with any IOP value by the manufacturer (Figure 1).

In Michaud's study, numerical gradations were added to the scale at 10, 15, 20, 25, and 30 mmHg by Ingeneus to provide semi‐quantitative guidance (Figure 1).

**FIGURE 2:**
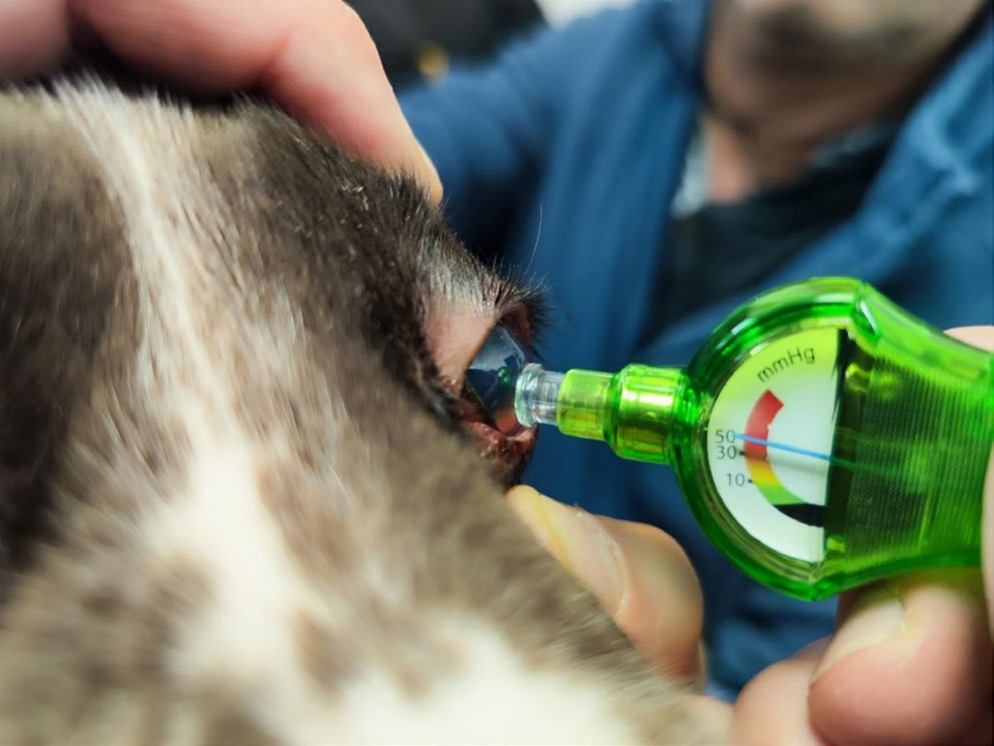
Intraocular pressure (IOP) evaluation of the left eye in a 7‐year‐old English Springer Spaniel presented with unilateral primary glaucoma.

For the updated version of the IOPVet, we proposed the creation of a progressive color gradient ranging from green to red and recommended removing the upper black zone, which did not correspond to any physiologically or clinically relevant pressure range. Based on findings from Kapeller and Michaud's studies, three reference IOP values were explicitly marked: 10, 30, and 50 mmHg, with the 10 mmHg reference point remaining unchanged (Figures 1 and 2). These changes were intended to improve the usability and interpretability of the device by emphasizing its semi‐quantitative nature and by aligning the visual scale more closely with clinically relevant thresholds. While this redesign aimed to reduce user misinterpretation and facilitate early detection of ocular hypertension, the actual improvement in diagnostic accuracy was assessed through the present clinical and manometric validation.

While the simplified visual scale improves interpretability, the reduced spacing between markings may require proper training to avoid misclassification, especially at critical thresholds.

The aim of the present study was to evaluate the performance of a recalibrated version of the IOPVet tonometer for estimating intraocular pressure in a population of client‐owned dogs with confirmed glaucoma, and to assess its agreement with both a rTVP and direct manometry.

## 2 Material and Methods

### 2.1 Study Design and Animal Population

This prospective study was conducted at the Anima‐Vet Veterinary Clinic (France) and approved by the institutional ethics committee (VetAgroSup Lyon). Informed client consent was obtained for the use of all client‐owned animals included in this study. The study population included a total of 47 client‐owned dogs, divided into three distinct cohorts:

#### 2.1.1 Glaucomatous Group

The first cohort consisted of 34 dogs (Table 1) of various breeds, sexes, and ages diagnosed with either primary or secondary glaucoma determined by complete ophthalmological examination and an intraocular pressure (IOP) higher or equal to 30 mmHg measured with the rTVP. Among them, 7 dogs were bilaterally affected, resulting in a total of 41 glaucomatous eyes. Cases were classified as either primary (*n* = 10 eyes; 24.4%) or secondary glaucoma (*n* = 31 eyes; 75.6%). Among the secondary cases, the underlying causes included lens luxation (*n* = 13 eyes; 41.9%), uveitis (*n* = 7 eyes; 22.6%), cataract (*n* = 6 eyes; 19.4%), and neoplasia (*n* = 5 eyes; 16.1%). This heterogeneous population reflected a realistic perspective for validating the device's accuracy under practical clinical conditions.

**TABLE 1:** Breeds represented in the study population overall.

Breed	*N*	Percent
Jack Russell Terrier	8	16.33%
French Bulldog	5	10.20%
Siberian Husky	5	10.20%
Chihuahua	3	6.12%
Shih Tzu	3	6.12%
Brittany Spaniel	2	4.08%
Maltese	2	4.08%
Pekingese	2	4.08%
Belgian Shepherd	2	4.08%
Other breeds	17	34.69%
Total	49	100%

The mean age of the dogs in this group was 10.12 years (±3.99 years). Among them were 21 males (5 neutered and 16 intact) and 13 females (2 intact and 11 spayed).

A total of 41 eyes were classified as glaucomatous, comprising 20 right eyes and 21 left eyes, with 7 dogs presenting bilateral involvement. The 26 fellow eyes of these dogs, which showed no clinical signs and IOP under 30 mmHg, were included as internal controls for comparison.

#### 2.1.2 Clinically Normal Control Group

The second cohort consisted of 10 clinically normal dogs presented to the preventive medicine service of the same clinic, with no history or clinical signs of ocular disease. These dogs of diverse breeds were evaluated during routine health checks or pre‐anesthetic assessments. The mean age of this group was 4.80 years (±3.88 years). The population included three intact males, two neutered males, three intact females, and two spayed females.

#### 2.1.3 Manometry Group

The third cohort included five dogs euthanized at the clinic for non‐ophthalmic reasons. These animals had no known history or clinical signs of ocular pathology. The dogs were of five different breeds: Griffon Nivernais, Jack Russell Terrier, Siberian Husky, Boston Terrier, and Belgian Shepherd. Their mean age was 13.2 years (±3.49 years). The group included two males and three spayed females.

### 2.2 Instrumentation and Measurement Evaluation

All IOP measurements were conducted in a quiet examination room, with dogs gently restrained in a sitting or standing position. Special care was taken to avoid any pressure on the neck or the globe during the procedure. For all animals except euthanized dogs, IOPs were first assessed in mmHg with the rTVP by the first examiner (ID) using the “dog” calibration mode. Six consecutive readings were obtained, and the device automatically calculated the final value by averaging the central measurements and discarding outliers. The probe was systematically replaced for each dog to ensure hygiene and consistency. Then a drop of oxybuprocaine 0.4% (Cébésine—Laboratoire Chauvin, France) was instilled to achieve consistent corneal anesthesia prior to estimating IOP by the second examiner (BM) using a sterile recalibrated IOPVet for each patient.

Both operators were masked to each other's results. For each single dog, the first eye to be assessed was chosen randomly to avoid any systematic bias. The IOPVet device was applied perpendicularly to the central cornea. The spring‐loaded plunger was gently depressed to obtain a visible reading, taking care not to cause any posterior displacement of the globe into the orbit (Figure 2). For each eye, the estimates obtained with the IOPVet were recorded along with the pressure interval it belonged to. These intervals were defined as follows: pressures below 10 mmHg, pressures between 10 and 29 mmHg, those between 30 and 49 mmHg, and pressures equal to or above 50 mmHg.

**FIGURE 3:**
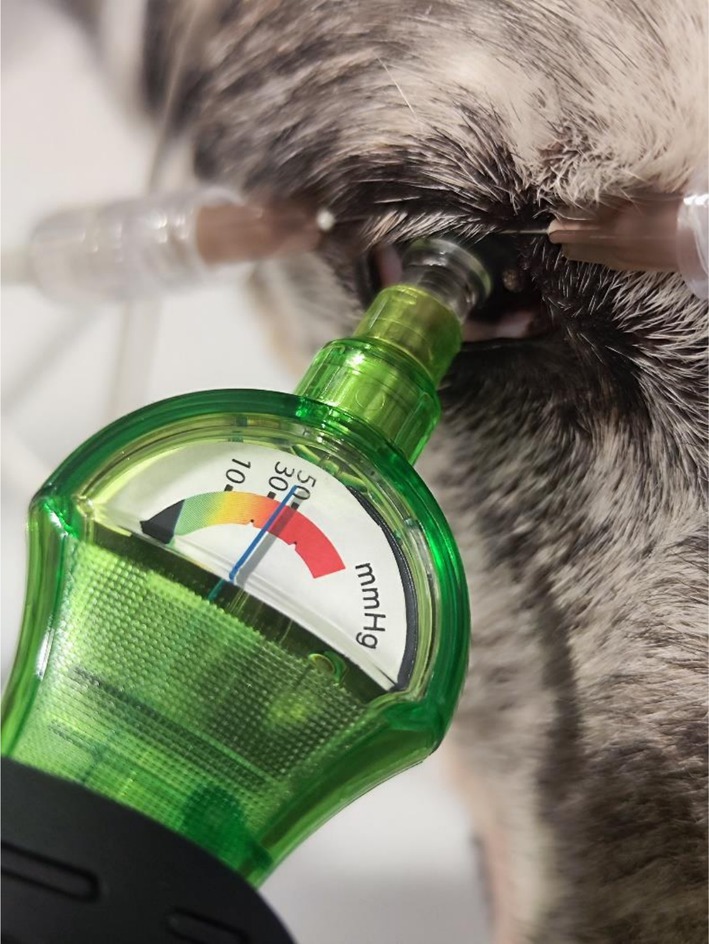
IOP evaluation with the IOPVet after in situ cannulation of the eye through the cornea using two 26‐gauge 5/8‐in. needles: one connected to a fluid column and the other to a manometer.

**FIGURE 4:**
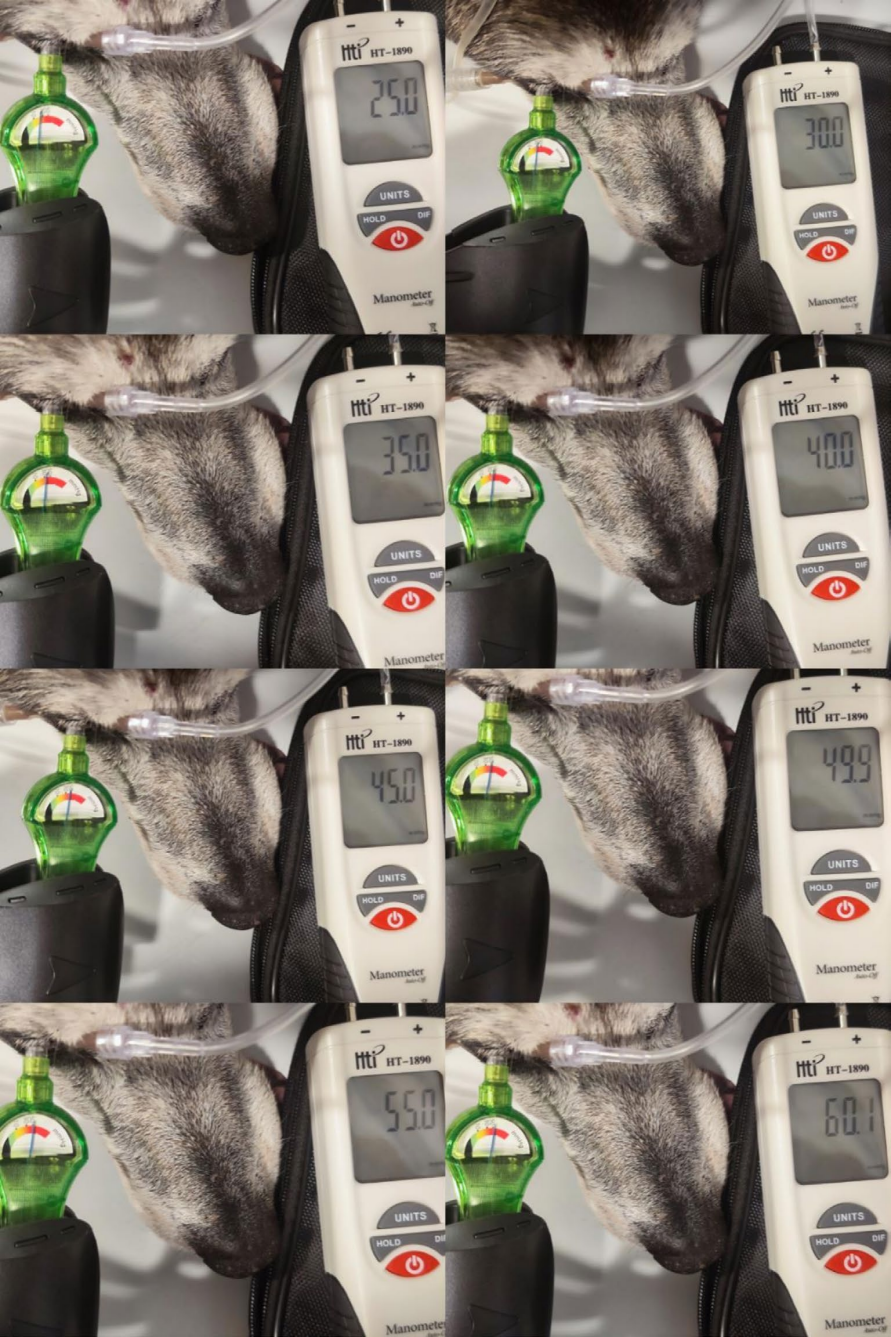
IOP evaluation with the IOPVet and by manometry for pressures ranging from 25 to 60 mmHg, in 5 mmHg increments.

To validate the recalibrated IOPVet against real intraocular pressure, a manometric reference technique was employed in situ after euthanasia. Each eye was cannulated through the cornea using two 26‐gauge 5/8‐in. needles: one connected to a fluid column, and the other to the manometer (HT‐1890 Manometer, HTI Instrument, Dongguan, China) (Figure 3). The dual 26G needles were inserted obliquely through the peripheral cornea at the 2 and 10 o'clock meridians, approximately 1 mm from the limbus. No leakage was observed at any cannulation site. This setup enabled precise control and stepwise adjustment of intraocular pressure from 10 to 70 mmHg in 5 mmHg increments (Figure 4). The digital was a new device, factory‐calibrated and tested prior to the start of the study, ensuring accuracy and consistency across all pressure measurements.

Contrary to conventional ex vivo validation protocols involving enucleated eyes, the globes remained in place to preserve the anatomical and biomechanical integrity of the eye and its surrounding tissues, hence reflecting more accurately the in vivo conditions of indentation tonometry. A custom fixation system was used to maintain the IOPVet in a stable, perpendicular position against the central cornea, ensuring constant applanation force and eliminating operator‐dependent variability. The device was initially calibrated by setting the manometric pressure to 10 mmHg and verifying its correspondence on the IOPVet scale. Manometric readings were considered acceptable if they remained within ±0.2 mmHg of the target pressure before IOPVet estimation was performed. Throughout the session, the corneal surface was regularly moistened with sterile saline solution to maintain hydration and preserve tissue biomechanics. All measurements were performed on 10 eyes from the five euthanized dogs within 1 h after death. All procedures adhered to the ARVO Statement for the Use of Animals in Ophthalmic and Vision Research and complied with the GERVO.

### 2.3 Data Analysis

All statistical analyses were performed using R software (version 4.3.1) and SPSS software (version 29). Descriptive statistics (mean ± standard deviation) were calculated for continuous variables such as rTVP and manometric values. Given the semi‐quantitative, categorical nature of the IOPVet readings, agreement between devices was assessed using 4 × 4 contingency tables, sensitivity and specificity analyses, and Chi‐square tests. Pressure ranges were predefined as < 10, 10–29, 30–49 mmHg, and ≥ 50 mmHg. The IOPVet's classification performance was compared against reference values obtained from rTVP (in vivo) and manometry (in situ). This categorical analytical approach was selected as the most appropriate given the ordinal output provided by the IOPVet device.

## 3 Results

A total of 93 eyes from 47 client‐owned dogs were included. Forty‐one eyes were diagnosed with glaucoma based on clinical findings and a rTVP‐measured intraocular pressure (IOP) ≥ 30 mmHg. The remaining 46 eyes (26 contralateral clinically normal eyes from glaucomatous dogs and 20 eyes from healthy control dogs) were considered normotensive (rTVP ≤ 29 mmHg). Ten eyes from five euthanized dogs were used for manometric validation.

### 3.1 Agreement Between Recalibrated IOPVet and rTVP

Among glaucomatous eyes (rTVP ≥ 30 mmHg, *n* = 41), all were classified in elevated pressure ranges by the recalibrated IOPVet. Seventeen eyes (41.5%) were estimated in the 30–49 mmHg range and 24 eyes (58.5%) in the ≥ 50 mmHg range. For eyes with rTVP IOP measurement between 30 and 49 mmHg (*n* = 20), 16 (80.0%) were correctly categorized by the IOPVet, while four (20.0%) were overestimated. For eyes with rTVP IOP ≥ 50 mmHg (*n* = 20), 19 (95.0%) were correctly classified.

**FIGURE 5:**
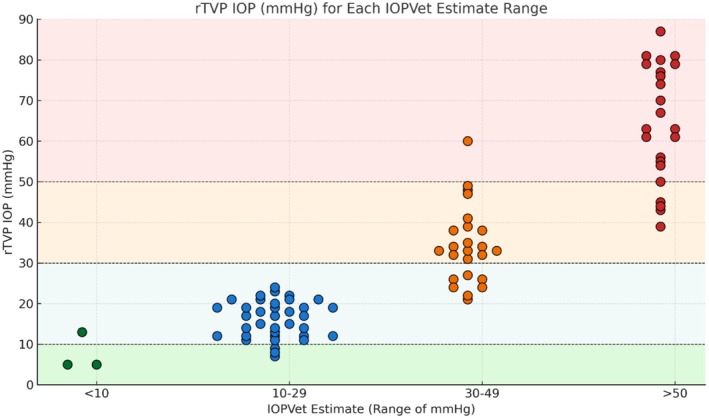
In vivo comparison of IOPVet estimates (horizontal axis) with rTVP intraocular pressure (IOP) values (vertical axis, mmHg). The shaded background areas represent the expected location of each data point if the IOPVet estimate was correct: Light green for IOP < 10 mmHg, light blue for IOP between 10 and 29 mmHg, light orange for IOP between 30 and 49 mmHg, and light red for IOP ≥ 50 mmHg. Each dot represents a single eye, with color intensity and grouping reflecting the IOPVet‐estimated range. Multiple dots aligned horizontally at the same pressure indicate repeated occurrences of that IOP value.

In normotensive eyes (rTVP ≤ 29 mmHg, *n* = 46), the IOPVet estimated 3 eyes (6.5%) as < 10 mmHg, 37 eyes (80.4%) as 10–29 mmHg, and 6 eyes (13.0%) were overestimated (classified as 30–49 mmHg or ≥ 50 mmHg). The IOP estimations with the IOPvet and correlations with the TVP measurements are presented in Figure 5.

The resulting specificity was 87.0% and sensitivity was 100% for identifying IOPs ≥ 30 mmHg. The positive predictive value (PPV) was 87.0%, and the negative predictive value (NPV) was 100%.

Categorical agreement between rTVP and IOPVet readings was analyzed using a 4 × 4 contingency matrix. The Pearson's Chi‐squared test revealed a statistically significant association between IOPVet‐estimated categories and true rTVP measurements (χ^2^ = 205.2, df = 9, *p* < 0.0001), confirming that the IOPVet's categorizations were not due to chance.

The full distribution of IOPVet estimates across rTVP ranges is detailed in a confusion matrix in Table 2. Global sensitivity, specificity, and predictive values are also presented in Table 3.

**TABLE 2:** Confusion martrix number of IOPvet estimates compared to rTVP IOPs.

rTVP IOP (mmHg)	IOPVet estimates (mmHg)			
	< 10	10–29	30–49	≥ 50
< 10	2	3	0	0
10–29	1	33	7	0
30–49	0	0	16	4
≥ 50	0	0	1	20

*Note:* Light green corresponds to hypotensive values (< 10 mmHg), light blue to normal‐to‐borderline values (10–29 mmHg), light yellow to moderate ocular hypertension (30–49 mmHg), and light red to severe ocular hypertension (≥ 50 mmHg).

**TABLE 3:** Sensitivity, specificity, and predictive values with 30 mmHg threshold.

Glaucoma (> 30 mmHg)	Normal (≤ 30 mmHg)	Predictive values
True affected: 41	False affected: 7	Positive: 85%
False normal: 0	True normal: 39	
Sensitivity 100%	Specificity 85%	Negative: 100%

### 3.2 Manometric Validation (In Situ)

**FIGURE 6:**
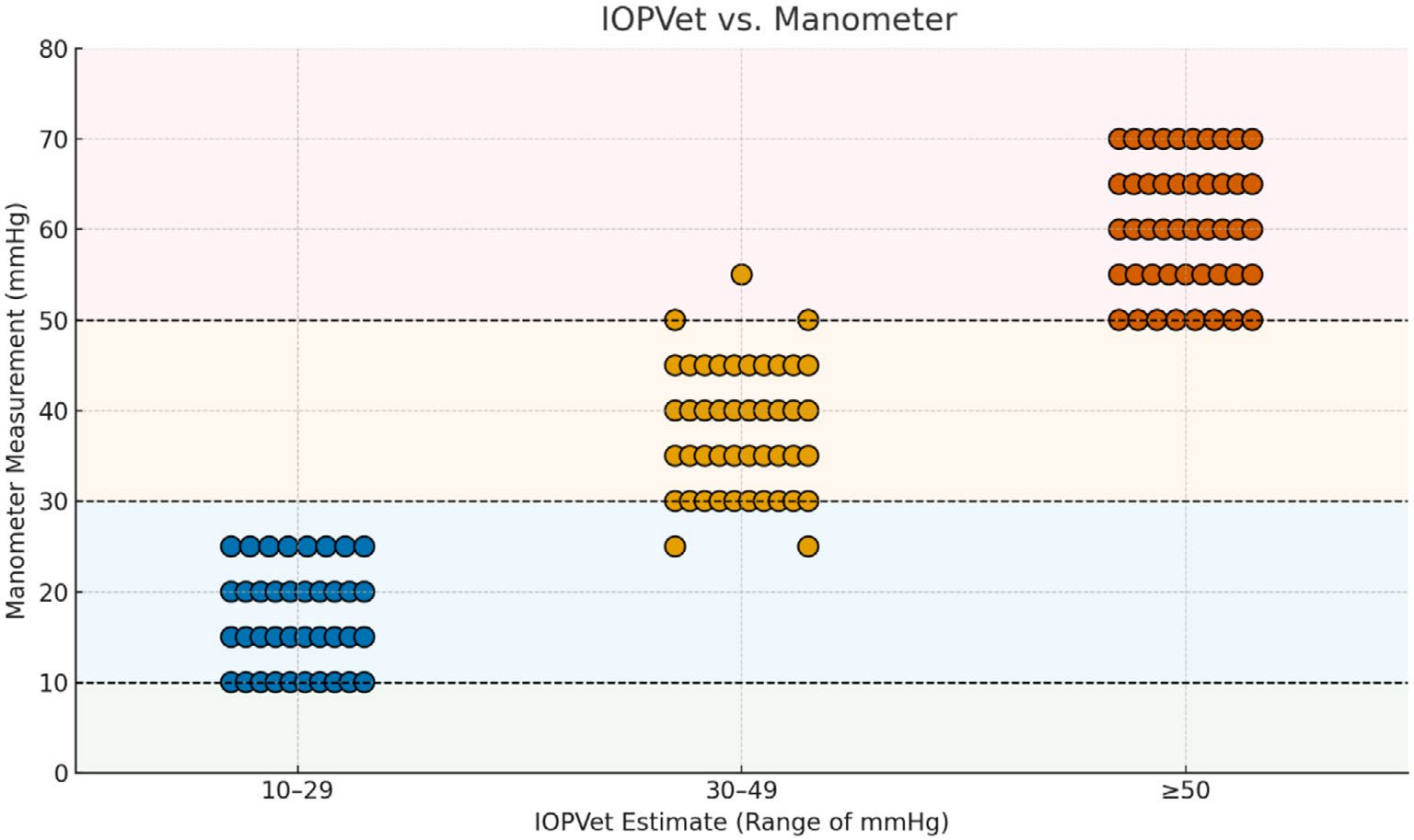
In situ comparison of IOPVet estimates (horizontal axis) with manometric intraocular pressure (IOP) values (vertical axis), from 0 to 80 mmHg. Colored background zones show the expected position of the points if the IOPVet estimate was correct: Green for 0–10 mmHg, blue for 10–29 mmHg, orange for 30–49 mmHg, and red for ≥ 50 mmHg. Each dot represents one measurement, with dot color corresponding to the IOPVet‐estimated range.

Using a controlled in situ manometry protocol, a total of 130 intraocular pressure measurements were obtained across 10 eyes subjected to stepwise cannulation from 10 to 70 mmHg. Categorical agreement between the recalibrated IOPVet and true manometric values was high across all pressure ranges. In the interval (10–29 mmHg), 95% of readings (38/40) were accurately categorized. In the clinically critical range (30–49 mmHg), the device achieved 100% concordance (40/40), and in the ≥ 50 mmHg range, accuracy remained high at 94% (47/50). Crucially, no IOP measurement ≥ 30 mmHg was underestimated in a normotensive category. The IOPVet estimations and correlations with the manometric measurements are presented in Figure 6.

These results underscore the device's capacity to detect clinically relevant elevations in IOP under standardized experimental conditions. The absence of false negatives above the diagnostic threshold supports the reliability of the recalibrated IOPVet for identifying pathologic ocular hypertension in a controlled setting.

## 4 Discussion

The present study offers a robust evaluation of a recalibrated version of the IOPVet indentation tonometer for use in dogs, combining both in vivo clinical assessments and in situ manometric validation. In contrast to previous reports that highlighted significant limitations in the accuracy of the original IOPVet model, our data support the clinical utility of the updated version, particularly for detecting elevated intraocular pressure (IOP) in glaucomatous patients. The refinement of the device's scale appears to have markedly improved its accuracy and diagnostic value.

Our results stand in clear contrast with earlier studies using the first‐generation IOPVet. Kapeller et al. reported a sensitivity as low as 13% for detecting IOPs > 30 mmHg, with only 16 of 125 glaucomatous eyes correctly identified [1]. Likewise, Michaud and Lesne found poor sensitivity (33.3%) for high IOPs, despite good specificity for normotensive values [2]. These limitations had cast doubt on the device's clinical reliability. It should be noted, however, that while our findings highlight a significant improvement compared to earlier reports using the previous IOPVet model, the two versions differ in their visual scale design and the classification thresholds applied. As such, diagnostic sensitivity and specificity values obtained in these respective studies may not be directly comparable. Any apparent improvement in performance must therefore be interpreted in the context of both design modifications and revised pressure intervals. However, both studies had methodological constraints, and their findings underscored the importance of recalibration and standardized protocols—key elements in the improved performance observed in our study.

Kapeller's work, for instance, was conducted exclusively on a homogeneous population of purpose‐bred Beagles with hereditary open‐angle glaucoma, a model that does not reflect the clinical variability encountered in general practice. In contrast, our study included a heterogeneous population of client‐owned dogs with both primary and secondary glaucoma, spanning a wide range of breeds, ages, and clinical presentations. This diversity strengthens the external validity of our findings and supports the device's relevance for everyday clinical practice.

Similarly, the study by Michaud and Lesne did not include any manometric validation, limiting the ability to assess the true accuracy of the device. Without direct intraocular pressure measurements as a reference, performance estimates may be subject to bias.

In our clinical evaluation, the recalibrated IOPVet demonstrated a sensitivity of 100% for detecting eyes with an IOP ≥ 30 mmHg, and a specificity of 87.0% for values ≤ 29 mmHg when compared to the recalibrated TonoVet Plus (rTVP).

None of the glaucomatous eyes were erroneously categorized into a normotensive range by the IOPVet, a major improvement over previous performance reports. In normotensive eyes, only 7 cases were overestimated in higher categories, an acceptable margin given the semi‐quantitative nature of the device.

Our study also incorporated an in situ manometric validation component. Unlike ex vivo studies conducted on enucleated globes, which eliminate the influence of periocular structures and orbital support, our in situ manometric approach was designed to replicate the in vivo anatomical context as closely as possible [3, 5]. This methodology aimed to preserve the natural biomechanical resistance of the globe within the orbit, which can influence the accuracy of indentation‐based measurements [4, 5]. In a seminal study on rhesus monkeys, Eisenlohr et al. (1962) demonstrated that the pressure–volume relationship of the eye is significantly altered following enucleation, with increased ocular compliance and reduced resistance to deformation [6]. These findings highlight the potential for orbital and periocular tissues to affect intraocular pressure dynamics [3, 5]. By maintaining orbital integrity, our setup allowed for a more physiologically relevant validation of the IOPVet tonometer, particularly in the context of its intended clinical use. Manometry was performed in situ within 1 h after euthanasia, with continuous corneal hydration, using a dual‐needle cannulation setup as described in previous comparative studies [3, 5]. The recalibrated IOPVet showed strong concordance with manometric pressures across all the ranges tested. More specifically, 95% of readings in the 10–29 mmHg range, 100% in the 30–49 mmHg range, and 94% in the ≥ 50 mmHg range were correctly categorized. These results show the IOPVet's greater reliability for detecting clinically significant elevations in intraocular pressure, particularly above the 30 mmHg threshold where diagnostic accuracy is most critical.

The improved performance of the IOPVet is likely multifactorial. First, the removal of the uninformative upper black zone from the scale, which in the original model did not correspond to any physiological range, likely reduced user confusion. Second, the introduction of a gradual color gradient, replacing the sharply defined green‐yellow‐red blocks, will help users appreciate the continuous nature of IOP variation. Third, numerical indicators at 10, 30, and 50 mmHg offer important landmarks for semi‐quantitative interpretation. These changes, based on direct clinical feedback and prior data, appear to have significantly enhanced the device's diagnostic reliability.

Importantly, while the IOPVet remains a semi‐quantitative device, it does not rely on complex electronics, requires no calibration, and is disposable. Applanation devices like the Tonopen XL and Schiötz tonometer, which in the past were used for similar indications, suffer from inter‐operator variability, corneal sensitivity requirements, and have been largely supplanted by rebound devices [7, 8]. As with applanation‐based devices such as the Tonopen Avia, which are known to be significantly influenced by corneal biomechanical properties—particularly in glaucomatous eyes with altered stromal rigidity [4, 5]—the IOPVet may also be subject to the effects of central corneal thickness (CCT), despite its reliance on indentation mechanics. This potential influence was not assessed in our study and represents a relevant limitation to be addressed in future evaluations.

Nevertheless, we acknowledge several limitations: the number of glaucomatous dogs could have been higher to improve the accuracy of our study. The absence of pachymetry in our protocol constitutes a methodological weakness. While prior studies have shown that CCT can affect readings obtained with applanation and rebound tonometers [4, 9], its influence on indentation‐based estimations remains unclear. Incorporating pachymetry in future work would allow for correction factor calculations and more accurate comparisons. Another limitation is the relatively small size of the manometric cohort, which, although methodologically robust, included only 10 eyes from five dogs. Although our results were consistent and the in situ technique minimized experimental artifacts, a larger sample would enhance statistical confidence.

Another main consideration is user variability. Although the IOPVet is designed for simplicity, its operation still requires correct positioning, steady pressure application, and accurate reading interpretation. These factors may introduce subjectivity, particularly in the hands of untrained individuals. Although the simplified design of the device may theoretically allow at‐home IOP monitoring in the future, its current use should remain strictly veterinary‐guided, as it requires corneal anesthesia and appropriate operator training. Glaucoma management often necessitates frequent pressure checks, and acute IOP spikes may go unnoticed without home surveillance. Devices like the IOPVet could empower owners of glaucomatous dogs to detect early signs of relapse and alert veterinarians promptly. While such practice raises concerns about misinterpretation or false alarms, it holds promise for improving patient outcomes, especially in chronic cases or in remote areas lacking specialist access.

Finally, the scope of the IOPVet may extend beyond canine applications. The demand for cost‐effective, user‐friendly tonometers is equally strong in feline ophthalmology, where stress‐induced IOP elevations can confound clinical interpretation, and in equine medicine, where equipment portability and resilience are critical. Several recent papers have explored the use of rebound tonometers in cats and rabbits [10], but no evaluation of the IOPVet has yet been undertaken in these species. Given the anatomical and biomechanical differences among species, such studies are necessary before broader clinical recommendations can be made.

## 5 Conclusion

In conclusion, our findings support the clinical relevance of the recalibrated IOPVet for the semi‐quantitative estimation of IOP in dogs, particularly in the context of glaucoma screening and monitoring. Its affordability, accuracy, and usability represent a significant improvement over previous iterations and justify its integration into clinical practice, especially where access to more expensive devices is limited. While not intended to replace digital tonometry, it may serve as a valuable adjunct as a complementary tool for veterinary use. While the IOPVet's simplicity may suggest potential for home‐based applications in the future, such use remains ethically sensitive and requires careful veterinary supervision due to the need for topical anesthesia and proper user training. Further studies addressing pachymetric variability, expanding manometric validation, and exploring multispecies applications will help refine its role in the expanding landscape of veterinary ophthalmic diagnostics.

## Ethics Statement

All procedures involving animals were approved by the Animal Ethics Committee of VetAgro Sup, Lyon, France (approval number: AEC 2023–042). Written informed consent was obtained from all dog owners prior to inclusion in the study. The research was conducted in full compliance with European Directive 2010/63/EU and the ARRIVE guidelines.

## Conflicts of Interest

We proposed a new scale for the IOPVet to Ingeneus, which agreed to develop it and provided the devices necessary to carry out our study. No financial compensation or influence on data analysis or interpretation occurred.

## Data Availability

The data that support the findings of this study are openly available in Dataset—Validation of Recalibrated IOPVet in Canine Eyes (https://www.doi.org/10.6084/m9.figshare.29042771).
